# Cross-Species Insights Into Genomic Adaptations to Hypoxia

**DOI:** 10.3389/fgene.2020.00743

**Published:** 2020-07-22

**Authors:** Matthew E. Pamenter, James E. Hall, Yuuka Tanabe, Tatum S. Simonson

**Affiliations:** ^1^Department of Biology, University of Ottawa, Ottawa, ON, Canada; ^2^Ottawa Brain and Mind Research Institute, University of Ottawa, Ottawa, ON, Canada; ^3^Division of Pulmonary, Critical Care, and Sleep Medicine, Department of Medicine, School of Medicine, University of California, San Diego, San Diego, CA, United States

**Keywords:** hypoxia, high-altitude adaption, Tibetan, Andean, Ethiopian, EPAS1, HIF pathway, genomic adaptations

## Abstract

Over millions of years, vertebrate species populated vast environments spanning the globe. Among the most challenging habitats encountered were those with limited availability of oxygen, yet many animal and human populations inhabit and perform life cycle functions and/or daily activities in varying degrees of hypoxia today. Of particular interest are species that inhabit high-altitude niches, which experience chronic hypobaric hypoxia throughout their lives. Physiological and molecular aspects of adaptation to hypoxia have long been the focus of high-altitude populations and, within the past decade, genomic information has become increasingly accessible. These data provide an opportunity to search for common genetic signatures of selection across uniquely informative populations and thereby augment our understanding of the mechanisms underlying adaptations to hypoxia. In this review, we synthesize the available genomic findings across hypoxia-tolerant species to provide a comprehensive view of putatively hypoxia-adaptive genes and pathways. In many cases, adaptive signatures across species converge on the same genetic pathways or on genes themselves [i.e., the hypoxia inducible factor (HIF) pathway). However, specific variants thought to underlie function are distinct between species and populations, and, in most cases, the precise functional role of these genomic differences remains unknown. Efforts to standardize these findings and explore relationships between genotype and phenotype will provide important clues into the evolutionary and mechanistic bases of physiological adaptations to environmental hypoxia.

## Introduction

Some of the most challenging environments for terrestrial life are those in which oxygen availability is limited. For terrestrial vertebrates, such hypoxic environments occur in high-altitude niches, where ambient oxygen levels are reduced relative to sea level due to lower barometric pressure, and in underground burrows, wherein animal respiration combined with poor gas diffusion through the surrounding soils results in decreased oxygen availability. A key distinction between these environments is that hypoxia is constant at high-altitude, whereas in underground burrows, hypoxia varies both in its intensity and consistency. Despite the challenges that hypoxia imposes on physiology, humans have inhabited hypoxic high-altitude regions for 10, 000 of years, while other mammals have evolved to adapt to life under conditions of hypoxia over millions of years or were brought to high altitudes via human intervention (e.g., domesticated animals) within a few hundred to several 1000 years ago (Qi et al., 2013; Witt and Huerta-Sanchez, 2019). Therefore, it is possible to explore common physiological and genetic adaptations to hypoxia across varied and distinct time spans and environmental conditions in both human and animal populations.

**FIGURE 1:**
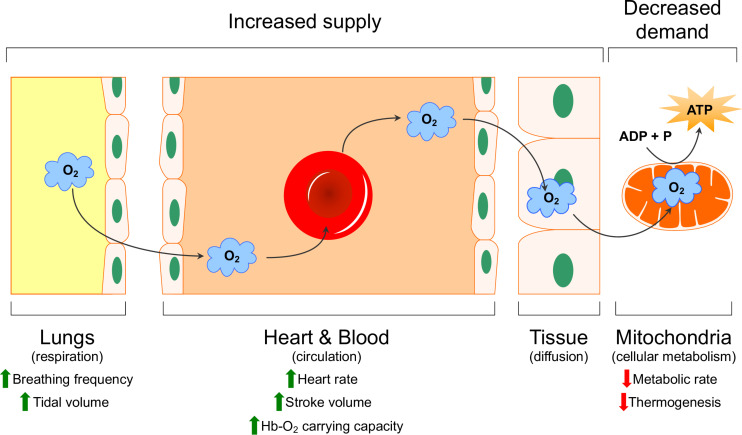
Schematic representation of the oxygen transport cascade. Physiological mechanisms that increase oxygen supply (green arrows) and decreased demand (red arrows) are indicated. Modified with permission from Dzal et al. (2015).

Limited oxygen availability is one of the strongest drivers of evolutionary adaptation and has resulted in the appearance of a wide variety of adaptive strategies. Over the past several decades, much effort has gone into characterizing physiological and molecular adaptations to hypoxia in various species adapted to life at high altitude or in underground burrows, and this work has been reviewed elsewhere (Hochachka, 1986; Hochachka et al., 1996; Bickler and Buck, 2007; Ramirez et al., 2007; Pamenter, 2014, 2016; Dzal et al., 2015; Pamenter and Powell, 2016; McClelland and Scott, 2019). These adaptations mitigate the impact of reduced environmental oxygen availability by enhancing its delivery through the body (i.e., increased supply) or, in situations where enhanced delivery is not sufficient, by reducing the need for oxygen at the tissue level to generate cellular energy (i.e., by decreasing metabolism; decreased demand) (Dzal et al., 2015; McClelland and Scott, 2019). The primary sites of adaptation in terms of oxygen supply reside within the cardio-respiratory system, whose primary function is to extract oxygen from the atmosphere and deliver it to the mitochondria of cells through four primary steps: ventilation, diffusion of oxygen from the air into the blood, circulation, and diffusion of oxygen from the blood into the cells (Figure 1). Common adaptations to hypoxia that have been observed in this oxygen transport cascade include changes in the sensitivity of chemosensory cells and organs to hypoxia, alterations in the anatomy, mechanics, and neural/cellular control of ventilation and cardiac function, biochemical changes to the oxygen carrying capacity of the blood, and anatomical adjustments that alter the diffusion distance across which gases must travel between the blood and tissues (and *vice versa*) (Dzal et al., 2015).

On the demand side of the hypoxia/energy balance reside systemic and cellular adaptations that minimize energy demand, either through reduced behaviors (i.e., torpor or hibernation), shutting down non-essential tissues to preserve energy for oxygen-sensitive organs, or by reducing energy demand at the cellular level by turning off specific processes (e.g., protein synthesis, thermogenesis, etc.) (Hochachka, 1986; Hochachka et al., 1996). Examining differences in the function and composition of the various components of the oxygen transport cascade and also cellular consumers of energy, and the control of these responses between species adapted to environments with variable oxygen availability has provided important insight into the evolution and function of oxygen-dependent processes.

Until somewhat recently, most studies have relied exclusively upon physiological and/or molecular approaches. While these studies have provided remarkable insight into the scope and variability of physiological responses and cellular adaptations to hypoxia, it is only with more recent genomic analysis of hypoxia-adapted human and animal populations that we have begun to understand the genetic underpinnings of these adaptations, their evolutionary origins, and, in a very few cases, links between genetic mutations and specific physiological and molecular characteristics.

In this review, we focus on the genomic aspects of hypoxia-adapted animals and humans from whom such information is available (regardless of the availability of matching physiological data) and identify overlap in common genes reported as top candidates for selection. The most commonly reported adaptation-associated genes are those related to the control and function of the hypoxia-inducible factor (HIF) pathway and/or downstream genes of this family of transcriptional regulators. This commonality has received considerable attention in comparative analysis of hypoxia-mediated genetic adaptation; however, many other genes have been identified across species, suggesting that there are multiple genetic avenues by which molecular and physiological adaptations to hypoxia might have evolved (Simonson, 2015). Adaptations in mammalian, bird, and ectothermic species that are native to high-altitude environments or that have been brought to high altitude as domesticated animals by humans, as well as species that experience hypoxia at low altitudes (e.g., mammals that live in densely populated underground burrows) are included for comparative purposes. There are many hypoxia-tolerant species in which physiological mechanisms of hypoxia-tolerance have been extensively described but for which genetic information is not available; discussion of these adaptations is outside the scope of the present review. However, many excellent reviews have been published recently that describe physiological adaptations to hypoxia and the interested reader is encouraged to consult these for further information (e.g., Bickler and Buck, 2007; Gilbert-Kawai et al., 2014; Dzal et al., 2015; Simonson, 2015; Moore, 2017).

## Hypoxia Signaling and the HIF Pathway

Hypoxia inducible factors (HIFs) are the master regulators of cellular response to hypoxia. These transcriptional factors are involved in many processes such as cellular metabolism, angiogenesis, erythropoiesis, regulation of bone and connective tissue development, and fetal development (Bigham and Lee, 2014). The HIF family of transcriptional factors is comprised of four protein orthologs, HIF-1α, HIF-2α, HIF-3α, and HIF-1β (Semenza, 2020). HIF-1α and HIF-1β are present and highly conserved among metazoans (Loenarz et al., 2011) and are expressed in all mammalian tissues (Wiener et al., 1996). HIF-2α and HIF-3α are found in vertebrates and are expressed in a cell-type specific manner (Semenza, 2020). HIF-1α has been shown to be essential to embryonic development (Cerychova and Pavlinkova, 2018), and HIF-2α is noted for regulation of *EPO* transcription, to produce erythropoietin (EPO) protein postnatally in the kidney (Gruber et al., 2007; Haase, 2013). While these transcription factors regulate thousands of genes, the roles of HIF-3α remain to be extensively characterized (Yang S. L. et al., 2015; Duan, 2016). The genes encoding the HIF proteins, *HIF1A* (HIF-1α), *EPAS1* (HIF-2α), and *HIF3A* (HIF-3α), each encode a number of protein isoforms via alternative splicing adding additional functional capabilities (Drutel et al., 2000; Gothié et al., 2000; Maynard et al., 2003; Dales et al., 2010; Pasanen et al., 2010; Heikkilä et al., 2011; Yang S. L. et al., 2015).

**FIGURE 2:**
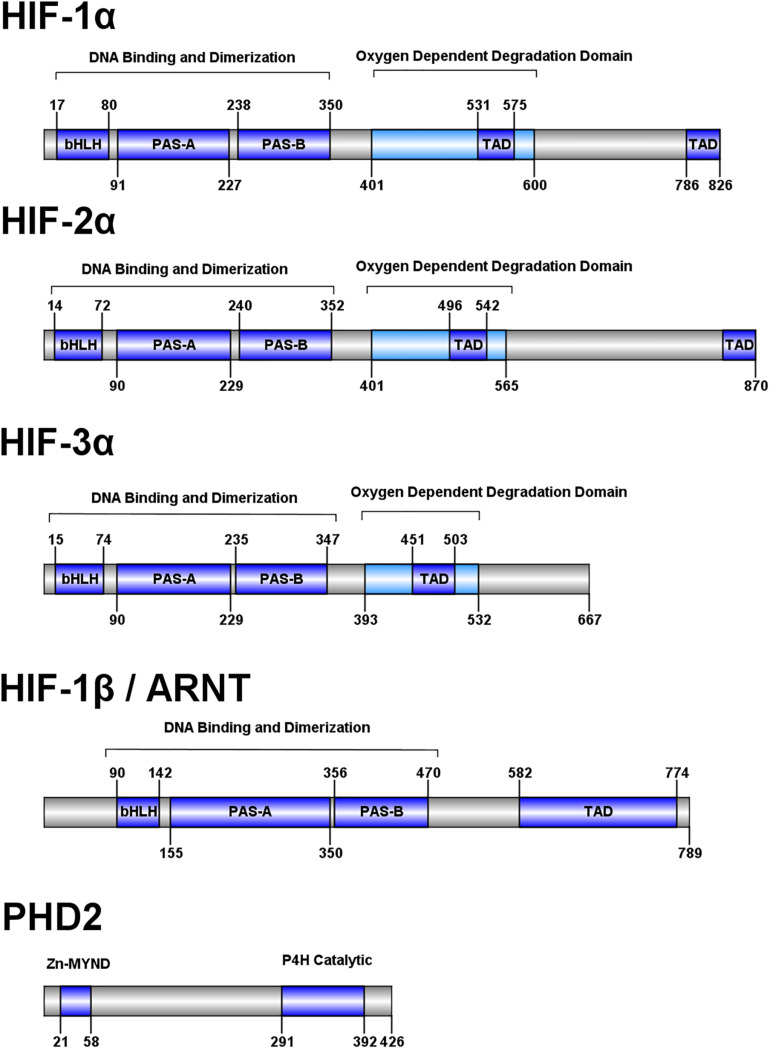
Domain structure of human HIF pathway proteins. Domain structure for HIF-1α, HIF-2α, HIF-3α, HIF-1β, and PHD2 are shown. The overall domain layout for the HIF proteins are similar with an N-terminal bHLH domain, followed by two PAS domains and an oxygen dependent degradation domain (absent in HIF-1β). HIF-1α and HIF-2α contain two transactivation domains in the C-terminal portion of the protein, while HIF-3α and HIF-1β have one. PHD2 has a domain layout containing an N-terminal MYND-type (myeloid, Nervy, and DEAF-1) zinc finger domain and a C-terminal prolyl-4-hydroxylase catalytic domain.

HIFs 1-3 share a conserved domain structure consisting of an N-terminal basic helix-loop-helix (bHLH) DNA binding domain, two Per-Arnt-Sim (PAS) domains, an oxygen-dependent degradation domain, and two transactivation domains (Figure 2; Beaudry et al., 2016). HIF-1β, also known as the aryl hydrocarbon nuclear translocator (ARNT) protein, shares some homology to HIFs 1-3 but lacks an oxygen-dependent degradation domain. The bHLH and PAS-A domains interact with DNA while the PAS domains are key for heterodimerization (Jiang et al., 1996; Wu et al., 2015). The oxygen-dependent degradation domain interacts with a number of regulatory proteins which modulate molecule stability and target the molecule for degradation (Metzen and Ratcliffe, 2004), and the transactivation domains recruit coactivators p300 and CREB-binding protein in the nucleus (Arany et al., 1996).

**FIGURE 3:**
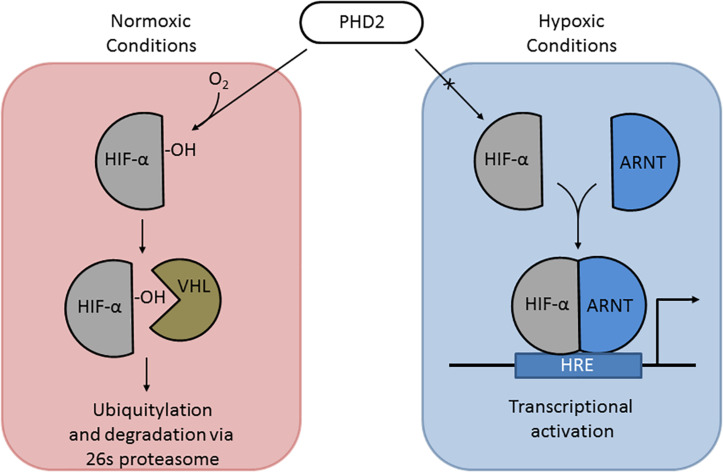
The HIF pathway. **(Left)** Under normoxic conditions, PHD2 hydroxylates proline 531 of HIF-2α, which can then be recognized by VHL, targeting it for ubiquitylation and degradation. **(Right)** Under hypoxic conditions, PHD2 cannot hydroxylate HIF α subunits, allowing it to be stabilized and translocate to the nucleus to dimerize with ARNT; the HIF-α-ARNT complex in the nucleus triggers transcriptional activation at hypoxia response elements (HREs).

Under normoxic conditions, HIF-1α and HIF-2α are regulated post-translationally via hydroxylation of specific proline residues within the oxygen-dependent degradation domain (Jaakkola et al., 2001; Figure 3). Hydroxylation is carried out by prolyl hydrolase domain (PHD) proteins PHD1-3 (Appelhoff et al., 2004). Hydroxylated HIF-α is recognized by von Hippel-Lindau (VHL) protein, which targets HIFs for degradation through the ubiquitin–proteasome pathway (Maxwell et al., 1999; Ivan et al., 2001). Under hypoxic conditions, this oxygen-dependent modification is arrested, leading to stabilization and accumulation of HIF-1α and/or HIF-2α within the cell (Taylor et al., 2016). HIF-1α and/or HIF-2α localize to the nucleus and dimerize with HIF-1β, which is required for DNA binding and transcriptional modulation (Ruas and Poellinger, 2005; Taylor et al., 2016). HIF-1 and HIF-2 complexes (HIF-1α/2α and HIF-1β dimer) recognize hypoxia response elements (HREs), which contain short conserved DNA sequences (5′-RCGTG-3′) (Semenza et al., 1996; Wu et al., 2015). HREs activate the expression of hypoxia-associated genes [e.g., *EPO*, *VEGF* (angiogenesis), *ALDOA*, *ENO1*, and *LDHA* (glycolytic enzymes) (Semenza et al., 1996)]. In addition to gene activation, HIF complexes can activate transcriptional repressors via the HIF target gene RE1-silencing transcription factor (REST) (Cavadas et al., 2016).

Genomic studies in search of adaptive signatures to high altitude indicate many HIF pathway genes and their downstream targets are top candidates for positive selection in humans (e.g., *EPAS1*, the gene the encodes HIF-2α and *EGLN1*, the gene that encodes PHD2) (reviewed in Bigham and Lee, 2014; Simonson, 2015). Studies in various high-altitude species have focused on these candidates and report specific variants, many of which are missense mutations and appear to be deleterious. It is interesting to note that while HIF-1α and HIF-2α have many overlapping and distinct targets, *HIF1A* does not repeatedly appear in selective sweeps like *EPAS1*. This may be due to the tendency of HIF mutations to be deleterious, HIF-1α playing a key role in embryonic development, and HIF-2α playing a major role in other processes with more plasticity.

### Investigating Structural Variations Among Species Within EPAS1

**FIGURE 4:**
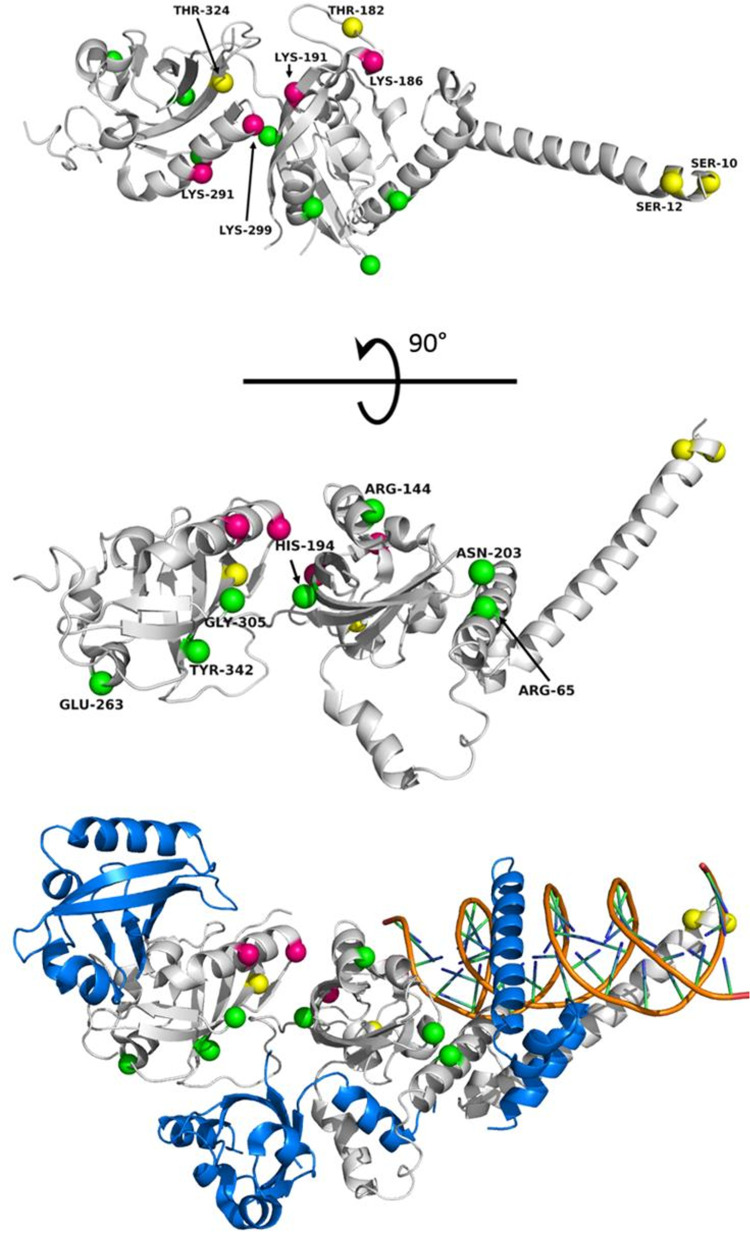
Known *EPAS1* variants mapped to the structure of the DNA binding and dimerization domain of HIF-2α in various species. (Top) “Top” view of HIF-2α structure (PDB ID 4ZPK). (Middle) “Side” view of HIF-2α. Spheres representing locations of interest are mapped onto HIF-2α with magenta representing sites of ubiquitylation in humans (K186, K191, K291, and K299) (Akimov et al., 2018), yellow representing phosphorylation sites (S10, S12, T182, and T324), and green representing the locations of identified variants from genetic studies. The species, variation, and publication are listed in Table 1. (Bottom) “Side” view of HIF-2α bound to ARNT and DNA (PDB ID 4ZPK). HIF-2α is shown in gray. ARNT is shown in blue. The DNA double helix is shown in Orange. Note: Tyr333 in chickens maps to residue Tyr342 in humans.

Numerous variants have been identified within HIF-2α which have been associated with high-altitude adapted species and disease states (Table 1). With a number of crystal structures available for HIF-2α, PHD2, and VHL, structural analysis and insights into how alterations in protein sequence translate to perturbation of HIF pathway signaling are under investigation. The structure of the DNA binding and dimerization domain of HIF-2α (Residues 8-360) in complex with ARNT (Wu et al., 2015) is shown in Figure 4. Post-translational modification sites in HIF-2α that have been identified in humans cluster along the “top” of this domain while the majority of variants among species cluster along dimerization interfaces on the opposite side of the PAS domains. While little is known about how these mutations affect protein structure and function, predictive analysis and co-immunoprecipitation studies suggest mutations along the bHLH and PAS domains are typically disruptive in nature, leading to loss-of-function (Wu et al., 2015; VonHoldf et al., 2017). Understanding how these variants affect HIF-2α structure, domain dynamics, and protein−protein interactions with binding partners (e.g., ARNT and PHD2) are key for understanding the role HIF-2α structure plays in high-altitude adaptation and hypoxic signaling within the cell (Hall et al., 2020).

**TABLE 1:** Amino acid variants in *EPAS1* among species.

**Species**	**Region**	**Variation in *EPAS1***	**Disease State (if associated)**	**Predicted effect of variation**	**References**
Human	Andean	**H194R**			Eichstaedt et al., 2017
	Tibetan	**N203H**	Patent ductus arteriosus	Decreased transcriptional activity, enhanced interactions with PHD2 and VHL	Pan et al., 2018
		G724W	Ventricular septal defect		
	Patients with erythrocytosis	F374Y I533V P534L	Erythrocytosis	Reduction of hydroxylation by PHD2 and decreased recognition by VHL	Gardie et al., 2014
		M535T M535V M535I		Disruption of PHD2 interaction	
		G537W G537R D539E F540L		Reduction of hydroxylation by PHD2 and decreased recognition by VHL	
Horse	Tibetan	**R144C**		Gain-of-function, increased affinity for ARNT binding	Liu et al., 2019
		**G263D**			
Dog	Tibetan	**G305S**		Predicted destabilization of the PAS domain	Gou et al., 2014; Wang et al., 2014
		D494E V500M P750S			
Chicken	Tibetan	Y333C*			Li et al., 2017
Hot spring snake	Tibetan	**R65H**			Li J. T. et al., 2018
Beef cattle	United States	E270Q P362L A671G L701F A606T G610S	Pulmonary hypertension		Heaton et al., 2016
Cashmere goat	Tibetan	Q579L**		Reduction of hydroxylation by PHD2 and decreased recognition by VHL	Song et al., 2016

Variants in bold have been identified in high-altitude adapted studies and correspond to residues mapped on to the crystal structure in Figure 3. *Y333 corresponds to Y342 in humans and mice. **Q579 Corresponds to Q557 in humans.

Of the variants listed in Table 1, only a few have been functionally investigated *in vivo* to establish the effects upon hypoxic signaling. Within the PAS domain of HIF-2α, R144C has been identified as a gain-of-function adaptation in Tibetan horses (Liu et al., 2019), G305S has been identified in high-altitude dogs (Gou et al., 2014), and N203H has been associated with patent ductus arteriosus in Tibetan humans (Pan et al., 2018). The R144C variant was functionally investigated in human lung adenocarcinoma A549 cells, and this variation is reported to stabilize the PAS domain leading to increased ARNT binding. In the case of high-altitude dogs, G305 is highly conserved and invariant among other animals. Homology modeling and prediction of functional effects has suggested that the mutation of G305S may affect thermostability of the PAS domain and is most likely causal mutation for the *EPAS1* selective sweep in high-altitude dogs (Gou et al., 2014; Wang et al., 2014). The N203H variant has been linked to non-syndromic congenital heart disease in Tibetans, and functional studies suggest that this particular mutation not only shows decreased transcriptional activity but also results in enhanced protein−protein interactions with both PHD2 and VHL (Pan et al., 2018). Outside of the PAS domains, other identified mutations identified as gain-of-function in the HIF-2α gene are reported in patients with erythrocytosis (Gardie et al., 2014; Pan et al., 2018). These variations are found in the oxygen-dependent degradation domain, clustering around Proline 531, which can disrupt residue hydroxylation and/or recognition of HIF-2α by VHL (Furlow et al., 2009), leading to increased cellular levels of HIF-2α. Aside from general perturbation of protein structure and stability, these variations within the HIF-2α PAS domain highlight how amino acid substitutions can affect protein−protein interactions, post-translational modification, and transcriptional activation, leading to a myriad of adaptive or maladaptive results. These effects can further be perturbed through additional variation within PHD2 and VHL, which have been identified in disease states associated with erythrocytosis and several cancers (Gardie et al., 2014).

Recently, H194R, located at an intramolecular domain interface within HIF-2α that is also part of a binding groove for ARNT binding (Figure 4), was identified as a missense mutation in high-altitude Andeans (Eichstaedt et al., 2017). The location of the H194R mutation may play a role in not only the stability of the intramolecular domain interface of HIF-2α but also in ARNT binding. In addition to structural effects, H194R is adjacent to K191, which has been identified in proteomic studies as a ubiquitylation site in humans (Akimov et al., 2018). The proximity of a bulky positive residue next to this ubiquitylation site may interfere with ubiquitylation (Radivojac et al., 2010) via the VHL-associated E3 ubiquitin ligase complex, which would in turn lead to elevated levels of HIF-2α within the cell. While more functional investigation for variants within HIF-2α and PHD2 need to be conducted to determine specific contributions of the individual variations on hypoxic signaling, the information currently available on the locations and specific variations in *EPAS1* among different species showcase how this gene, and others, can provide many different avenues for affecting transcriptional regulation and physiological adaptation toward hypoxia.

## Human Studies

Human populations have resided for hundreds of generations in the Tibetan, Andean and Ethiopian highlands in Central Asia, South America, and East Africa, respectively. Over several millennia and despite challenges imposed by environmental hypoxia at altitudes greater than 3500 m, these populations have continuously inhabited and successfully passed down heritable traits necessary for survival. The Tibetan highlands have been continuously inhabited the longest, with a human presence noted 30, 000−40, 000 years before present (Zhang et al., 2018). Initial settlements of high-altitude regions in the Andes date to approximately the Terminal Pleistocene (∼ 12,000 years before present) shortly after the arrival of modern humans in South America (Rademaker et al., 2014), while populations have migrated in and out of the Ethiopian highlands for possibly the past 70, 000 years (Hassen, 1990). The unique sets of traits exhibited by highlanders today suggest distinct population histories have shaped the adaptive and maladaptive milieu in each population, although there is notable within-population variation in several key traits.

The key physiological traits exhibited by each continental population have been recently summarized (Gilbert-Kawai et al., 2014; Simonson, 2015; Moore, 2017; Bhandari and Cavalleri, 2019). On average, Tibetan and Ethiopian highlanders tend to exhibit hemoglobin concentrations that are lower relative to acclimatized sojourners and Andeans at high altitude (Beall, 2007). There is, however, substantial variation within these groups, and Andeans have increased incidence of excessive erythrocytosis and chronic mountain sickness (CMS) (Monge et al., 1989; León-Velarde et al., 2005; Corante et al., 2018). Many Andeans, relative to Tibetans, also exhibit a decreased hypoxic ventilatory response and minute ventilation (Beall et al., 1997) and experience further hypoxia challenges during sleep (Pham et al., 2017, Heinrich et al., 2020), which may exacerbate mal-adaptations in this population. In addition to these hallmark differences, there are notable distinctions across developmental stages and various physiological systems. A detailed list of gene names, protein names, and functions mentioned in this review can be found in Table 2. Efforts to examine the functional roles of this variation are necessary to unravel the complexities of adaptive and maladaptive physiological responses (Hall et al., 2020).

**TABLE 2:** Genes and associated protein names and functions.

**Gene name**	**Protein abbreviation**	**Protein name**	**Role/function**	**References**
*HIF1A*	HIF-1α	Hypoxia inducible factor 1α	Transcription factor	Wang et al., 1995
*EPAS1*	HIF-2α	Hypoxia inducible factor 2α; Endothelial PAS Domain Protein 1	Transcription factor	Liu et al., 2020
*HIF3A*	HIF-3α	Hypoxia inducible factor 3α	Transcription factor	Zhang P. et al., 2014
*ARNT*	HIF-1β / ARNT	Aryl Hydrocarbon Receptor Nuclear Translocator	Transcription factor	Hankinson, 2002
*EGLN2*	PHD1	Hypoxia-inducible factor prolyl hydroxylase 1	Prolyl hydroxylase	Schofield and Ratcliffe, 2004
*EGLN1*	PHD2	Hypoxia-inducible factor prolyl hydroxylase 2	Prolyl hydroxylase	Schofield and Ratcliffe, 2004
*EGLN3*	PHD3	Hypoxia-inducible factor prolyl hydroxylase 3	Prolyl hydroxylase	Schofield and Ratcliffe, 2004
*VHL*	VHL	Von Hippel–Lindau tumor suppressor	Tumor suppressor	Lerman, 2002
*EPO*	EPO	Erythropoietin	Hormone	Lacombe and Mayeux, 2008
*VEGF*	VEGF	Vascular endothelial growth factor	Signaling protein; angiogenic factor	Li, 2001
*ALDOA*	ALDOA	Aldolase, Fructose-Bisphosphate A	Plays a key role in glycolysis and gluconeogenesis. Catalyzes the reversible conversion of fructose-1, 6-bisphosphate to glyceraldehyde 3-phosphate (G3P) and dihydroxyacetone phosphate (DHAP)	Semenza et al., 1996
*ENO1*	ENO1	Enolase 1	Glycolytic enzyme. Catalyzes the conversion of 2-phosphoglycerate to phosphoenolpyruvate	Semenza et al., 1996; Park et al., 2016
*LDHA*	LDHA; LDH5	Lactate Dehydrogenase A	Catalyzes the inter-conversion of pyruvate and L-lactate with concomitant inter-conversion of NADH and NAD+	Semenza et al., 1996
*REST*	REST	RE1-Silencing Transcription factor	Transcriptional repressor	Liu et al., 2015
*PPARA*	PPARα; NR1C1	Peroxisome proliferator-activated receptor alpha	Transcription factor. A major regulator of lipid metabolism in the liver	Popeijus, 2018
*HBB*	HBB	Hemoglobin subunit beta	Protein molecule in red blood cells that carries oxygen	Antonini and Brunori, 1970
*HBG2*	HBG2	Hemoglobin subunit gamma	Protein molecule in red blood cells that carries oxygen (Normally expressed in the fetus)	Adachi et al., 1990
*HFE*	HFE	Human homeostatic iron regulator protein	Regulates circulating iron uptake	Enns, 2006
*PKLR*	PKLR	Pyruvate Kinase L/R	Catalyzes the production of pyruvate and ATP from phosphoenolpyruvate	Jarrar et al., 2020
*CYP17A1*	CYP17A1	Cytochrome P450 Family 17 Subfamily A Member 1	Steroid 17α-monooxygenase	Tan et al., 2017; Jarrar et al., 2020
*HMOX2*	HMOX2	Heme oxygenase 2	Cleaves heme to form biliverdin, which is converted to bilirubin by biliverdin reductase, and carbon monoxide	Yang et al., 2016b
*HLa-DQB1*	HLA-DQB1	Major histocompatibility complex, class II, DQ beta 1	Part of a cell surface receptor that plays a central role in the immune system through presenting peptides derived from extracellular proteins	Jones et al., 2006
*HLA-DPB1*	HLA-DPB1	Major Histocompatibility Complex, Class II, DP Beta 1	Part of a cell surface receptor that plays a central role in the immune system through presenting peptides derived from extracellular proteins	Jones et al., 2006
*ANKH*	ANKH	Progressive ankylosis protein homolog	Transmembrane protein that controls pyrophosphate levels	Zaka and Williams, 2006
*ZNF532*	ZNF532	Zinc Finger Protein 532	May function as a transcription factor	Alekseyenko et al., 2017
*COL4A4*	COL4A4	alpha4 (IV) chain of type IV collagen	Part of type IV collagen	Mariyama et al., 1994
*EDNRA*	EDNRA	Endothelin Receptor Type A	Human G protein-coupled receptor	Maguire and Davenport, 2015
*PRKAA1*	PRKAA1	Protein Kinase AMP-Activated Catalytic Subunit Alpha 1	Kinase	Seza et al., 2019
*VEGFB*	VEGFB	Vascular Endothelial Growth Factor B	Signaling protein; angiogenic factor	Li, 2001
*ELTD1*	ELTD1	EGF, latrophilin and seven transmembrane domain-containing protein 1	Involved in angiogenesis	Nechiporuk et al., 2001
*BRINP3*	BRINP3	BMP/Retinoic Acid Inducible Neural Specific 3	Inhibits neuronal cell proliferation by negative regulation of the cell cycle transition	Crawford et al., 2017
*NOS2*	NOS2	Nitric oxide synthase 2	Produces nitric oxide (NO)	Bigham et al., 2009
*TBX5*	TBX5	T-Box Transcription Factor 5	Transcription factor	Krishnan et al., 2008
*SENP1*	SENP1	SUMO Specific Peptidase 1	Catalyzes maturation of SUMO protein (small ubiquitin-related modifier)	Cheng et al., 2007; Krishnan et al., 2008
*ANP32D*	ANP32D	Acidic nuclear phosphoprotein 32 family member D	Tumor suppressor	Zhou et al., 2013
*FAM213A*	PRXL2A; PAMM; FAM213A	Peroxiredoxin-like 2 activated in M-CSF stimulated monocytes (PAMM); REDOX-regulatory protein FAM213A	Involved in redox regulation of the cell	Valverde et al., 2015
*SFTPD*	SFTPD	Surfactant Protein D	Innate immune system collectin	Ito et al., 2011
*GPR126*	GPR126; Adgrg6	Adhesion G protein–coupled receptor G6	G protein-coupled receptor	Cui et al., 2014
*THRB*	THRB	Thyroid Hormone Receptor Beta	Nuclear hormone receptor that can act as a repressor or activator of transcription	Otto and Fandrey, 2008
*EDNRB*	EDNRB	Endothelin receptor type B	G protein-coupled receptor	Stobdan et al., 2018
*BHLHE41*	BHLHE41	Basic Helix-Loop-Helix Family Member E41; SHARP1; DEC2	Transcriptional repressor	Azmi et al., 2003
*VAV3*	VAV3	Vav guanine nucleotide exchange factor 3	Guanine nucleotide exchange factor	Ulc et al., 2017
*RORA*	RORA; RORα; NR1F1	RAR-related orphan receptor A; RAR-related orphan receptor alpha; nuclear receptor subfamily 1, group F, member 1	Exchange factor for GTP-binding proteins RhoA, RhoG and, to a lesser extent, Rac1; involved in angiogenesis	Li H. et al., 2018
*CIC*	CIC	Capicua transcriptional repressor	Transcriptional repressor	Udpa et al., 2014
*LIPE*	LIPE	Lipase E, hormone-sensitive type	Hydrolyzes stored triglycerides to free fatty acids; converts cholesteryl esters to free cholesterol for steroid hormone production	Briançon-Marjollet et al., 2016
*PAFAH1B3*	PAFAH1B3	Platelet-activating factor acetylhydrolase 1B catalytic subunit 3	Inactivates paf by removing the acetyl group at the sn-2 position	Tjoelker and Stafforini, 2000
*SIRT7*	SIRT7	Sirtuin 7	NAD-dependent deacetylase	Hubbi et al., 2013
*RYR2*	RYR2	Ryanodine receptor 2	Calcium channel that mediates the release of Ca^2+^ from the sarcoplasmic reticulum into the cytoplasm	Liao et al., 2011
*ANGPT1*	ANGPT1	Angiopoietin 1	Signaling protein involved in angiogenesis	Liu et al., 2008
*ANGPTL4*	Angptl4	Angiopoietin-like 4	Serum protein that is involved with modulating triacylglycerol homeostasis	Zhu et al., 2012
*ADAM17*	ADAM17; TACE	ADAM Metallopeptidase Domain 17; tumor necrosis factor-α-converting enzyme	Cleaves the membrane-bound precursor of TNF-α to its mature soluble form	Chen et al., 2017
*ARG2*	ARG2	Arginase 2	Catalyzes the hydrolysis of arginine to ornithine and urea	Jiang et al., 2015
*FIGF*	FIGF; VEGF-D	C-fos-induced growth factor; vascular endothelial growth factor D	Growth factor active in angiogenesis	Morfoisse et al., 2015
*PGF*	PGF	Placental Growth Factor	Growth factor active in angiogenesis	Tudisco et al., 2017
*TMPRSS6*	TMPRSS6	Transmembrane Serine Protease 6; Matriptase-2	Serine protease which hydrolyzes a range of proteins including type I collagen, fibronectin, and fibrinogen	Maurer et al., 2012
*ADORA2A*	ADORA2A	Adenosine A2a Receptor	G protein-coupled receptor	Liu and Xia, 2015
*CCL2*	CCL2	C-C Motif Chemokine Ligand 2	Cytokine	Baay-Guzman et al., 2012
*ENG*	ENG	Endoglin	Role in the development of the cardiovascular system and in vascular remodeling	Tian et al., 2010
*PIK3C2A*	PIK3C2A	Phosphatidylinositol-4-Phosphate 3-Kinase Catalytic Subunit Type 2 Alpha	Generates phosphatidylinositol 3-phosphate (PtdIns3P) and phosphatidylinositol 3, 4-bisphosphate [PtdIns(3, 4)P2]	Alam et al., 2009
*ATP12A*	ATP12A	ATPase H^+^/K^+^ transporting non-gastric alpha2 subunit	Catalytic subunit of the ouabain-sensitive H^+^/K^+^ -ATPase that catalyzes the hydrolysis of ATP coupled with the exchange of H^+^ and K^+^ ions across the plasma membrane	Ge et al., 2013
*NOS3*	NOS3	Nitric Oxide Synthase 3	Produces nitric oxide (NO)	Ge et al., 2013
*ANGPTL3*	ANGPTL3	Angiopoietin-like protein 3	Secretory protein that regulates plasma lipid levels	Carbone et al., 2018
*PRCP*	PRCP	Lysosomal prolylcarboxypeptidase	Cleaves C-terminal amino acids linked to proline in peptides	Hagedorn, 2011
*SEC24B*	SEC24B	SEC24 Homolog B, COPII Coat Complex Component; Protein transport protein Sec24B	Part of the coat protein complex II (COPII) which promotes the formation of transport vesicles from the endoplasmic reticulum (ER)	Verma et al., 2018
*EPHA1*	EPHA1	Erythropoietin-Producing Hepatoma Receptor A1; EPH Tyrosine Kinase 1	Receptor tyrosine kinase	Gale and Yancopoulos, 1999
*MCAM*	MCAM; MUC18; CD146	Melanoma Cell Adhesion Molecule	Cell adhesion	Shih and Kurman, 1996
*KAT6A*	KAT6A	Lysine Acetyltransferase 6A	Histone acetyltransferase	Shukla et al., 2018
*MYH9*	MYH9	Myosin Heavy Chain 9	Appears to play a role in cytokinesis, cell shape, and cytoskeletal reorganization	Wang et al., 2019
*CYSLTR2*	CYSLTR2	Cysteinyl Leukotriene Receptor 2	G protein-coupled receptor	Shao et al., 2015
*MYLK*	MYLK	Myosin light chain kinase	Calcium/calmodulin-dependent myosin light chain kinase	Pandey et al., 2015
*SPINT1*	SPINT1	Serine Peptidase Inhibitor, Kunitz Type 1	Inhibitor of HGF activator and matriptase	Susen et al., 2019
*PPAP2B*	LPP3; PLPP3; PAP-2b; PPAP2B	Lipid phosphate phosphohydrolase 3; phospholipid phosphatase 3; phosphatidic acid phosphatase type 2B	Hydrolyzes extracellular lysophosphatidic acid (LPA) and short-chain phosphatidic acid	Ren et al., 2013
*STRA6*	STRA6	Signaling Receptor And Transporter Of Retinol STRA6	Retinol transporter	Shao et al., 2015
*SLC29A1*	ENT1	Equilibrative nucleoside transporter 1	Sodium-independent transporter for purine and pyrimidine nucleosides and for some nucleobases	Eltzschig et al., 2005
*MYO5B*	MYO5B	Myosin VB	May be involved in vesicular trafficking via its association with the CART complex	Shao et al., 2015
*AJUBA*	JUB; AJUBA	LIM Domain-Containing Protein Ajuba	Adapter/scaffold protein	Shao et al., 2015
*TGFBR3*	TGFBR3	Transforming Growth Factor Beta Receptor 3	Binds to TGF-α	Chu et al., 2011
*VASN*	VASN	Vasorin	May act as an inhibitor of TGF-beta signaling	Choksi et al., 2011
*ACAA2*	ACAA2	Acetyl-CoA Acyltransferase 2; 3-ketoacyl-CoA thiolase, mitochondrial	Catalyzes the last step of the mitochondrial fatty acid beta oxidation pathway	Foll et al., 2014
*PINK1*	PINK1	PTEN Induced Kinase 1		Lin et al., 2014
*SIRT1*	SIRT1	Sirtuin 1	NAD-dependent deacetylase	Chen et al., 2011
*SIRT2*	SIRT2	Sirtuin 2	NAD+ (nicotinamide adenine dinucleotide)-dependent deacetylase	Ekambaram and Parasuraman, 2017
*SOD2*	SOD2	Superoxide Dismutase 2	Converts superoxide into hydrogen peroxide and diatomic oxygen	van Patot and Gassmann, 2011
*NARFL*	NARFL; CIAO3	Nuclear Prelamin A Recognition Factor-Like Protein; Cytosolic Iron-Sulfur Assembly Component 3	Component of the cytosolic iron-sulfur protein assembly (CIA) complex; seems to negatively regulate the level of HIF1A expression	Huerta-Sánchez et al., 2013
*COX1*	COX-1; PTGS	Cyclooxygenase; prostaglandin-endoperoxide synthase	Key enzyme in prostaglandin biosynthesis	Gautier-Veyret et al., 2013
*ADAM9*	ADAM9	Disintegrin and Metalloproteinase Domain-Containing Protein 9	Cleaves and releases a number of molecules with important roles in tumorigenesis and angiogenesis	Ahmed et al., 2017
*ADAMTS9*	ADAMTS9	ADAM Metallopeptidase With Thrombospondin Type 1 Motif 9	Cleaves the large aggregating proteoglycans, aggrecan, and versican	Rodríguez-Manzaneque et al., 2015
*UBE2D1*	UBE2D1	Ubiquitin-conjugating enzyme E2 D1	Accepts ubiquitin from the E1 complex and catalyzes its covalent attachment to other proteins	David et al., 2010
*SRF*	SRF	Serum response factor	Transcription factor	Olson and Nordheim, 2010
*TXNRD2*	TXNRD2	Thioredoxin Reductase 2	Involved in the control of reactive oxygen species levels and the regulation of mitochondrial redox homeostasis	Yoshioka, 2015
*WNT7B*	WNT7B	Wingless-Type MMTV Integration Site Family, Member 7B	Ligand for frizzled family receptors; required for central nervous system (CNS) angiogenesis and blood-brain barrier regulation	Qu et al., 2013

### The Power of Detecting Selection in Human Genomes

Advances in high-throughput genomic technologies have provided unprecedented insight into population-specific patterns of variation, including signals indicative of genetic adaptations. The power of this approach was first illustrated in human data, whereby adaptive regions of the genome may be revealed in as few as 30 individuals (Pickrell et al., 2007). The extent of distinct and shared genetic adaptations and how these factors relate to the distinct suite of physiological traits exhibited within and across populations is an active area of research (Wuren et al., 2014; Jha et al., 2016; Cole et al., 2017).

### Human Genetic Adaptation to High Altitude: Himalayan Highlanders

The Tibetan Plateau is one of the largest and longest continuously inhabited high-altitude regions in the world, and its inhabitants are the most extensively studied human populations in terms of genetic adaptation to hypoxia. Some of the first genomic studies of Tibetans, revealed adaptive candidate genes involved in hypoxia sensing and response pathways (Beall et al., 2010; Bigham et al., 2010; Simonson et al., 2010; Yi Z. et al., 2010), including *EPAS1* and *EGLN1* and *PPARA* (*Peroxisome Proliferator Activated Receptor Alpha*); these genes were further associated with relatively lower hemoglobin concentration in Tibetans (reviewed in Simonson, 2015). Subsequent studies identified associations between the adaptive *PPARA* candidate gene and metabolic parameters suggesting reduced fatty acid oxidation (Ge et al., 2012), in addition to greater oxygen utilization and protection from oxidative stress in skeletal muscle (Horscroft et al., 2017; O’Brien et al., 2019). In addition to Tibetan populations, various studies across Sherpa and Central Asian populations provide further support of key adaptive signatures in these populations (Foll et al., 2014; Jeong et al., 2014; Arciero et al., 2018; Gnecchi-Ruscone et al., 2018).

### Shared Signatures of Adaptation Within and Across Himalayan Populations

Numerous studies, conducted across various locations within the Qinghai-Tibetan Plateau and Central Asia, corroborate many of the initial findings regarding key genetic adaptations (Bigham et al., 2010; Peng et al., 2011; Xu et al., 2011; Jeong et al., 2014; Wuren et al., 2014). In addition to *EPAS1* and *EGLN1* HIF-related genes, several non-HIF pathway targets have been reported in more than one independent study, including the hemoglobin gene cluster *HBB/HBG2*, *HFE*, *PKLR*, *CYP17A1*, and *HMOX2* (Simonson, 2015). A recent study across Himalayan populations provides additional gene candidates, including *HLa-DQB1/HLA-DPB1*, *ANKH*, *RPaa-384F7.2*, *AC068633.1*, *ZNF532*, and *COL4A4* (Yang et al., 2016a; Arciero et al., 2018). Despite tremendous progress on the genomics front, the precise functional variants that provide functional benefits in Tibetans and other populations remain largely unknown (Hall et al., 2020). One exception is at the *EGLN1* gene, whereby variants in the first exon found at high frequency in Tibetans (Asp4Glu; Cys127Ser) are reported to result in a gain of PHD2 function, which leads to increased HIF degradation under hypoxic conditions and erythroid progenitor disruption (Lorenzo et al., 2014). Other reports suggest these variants underlie a loss of PHD2 function via defective binding of co-chaperone p23 that would lead to increased HIF activity (Aggarwal et al., 2010; Song et al., 2014) and possibly ventilatory responses (Song et al., 2020).

### Archaic Mixture in Tibetans

Whole-genome sequencing of DNA from archaic populations, including Neanderthal and Denisovan individuals, has provided the opportunity to search for genetic admixture within the genomes of modern human populations. Some of the exchanged genetic material has proven adaptive over many generations and provide examples of what is termed adaptive introgression. One notable example of adaptive introgression occurred in Tibetans at the *EPAS1* gene locus, which exhibits one of the strongest adaptive signatures in Tibetans and is most similar to Denisovan DNA compared to DNA from other human populations (Huerta-Sánchez et al., 2014; Hu et al., 2017). Therefore, archaic genetic admixture provided variation that putatively helped Tibetans adapt to the high-altitude environment. This finding highlights the importance of understanding distinct population histories and unique genetic backgrounds in studies of genetic adaptation to high altitude. Interestingly, *EPAS1* is one of the genes reported most frequently as a candidate gene for high-altitude adaptation in other high-altitude species (Supplementary Table S1), and is further noted to exhibit a signature of adaptive introgression across species.

### Human Genetic Adaptation to High Altitude: Andean Highlanders

The first analysis of genome-wide adaptation in two Andean populations, the Quechua and Aymara, and subsequent comparison with Tibetans, concluded the genetic basis for altitude adaptation was dissimilar in the two continental populations, although an exception was noted at the *EGLN1* gene region (Bigham et al., 2010). Of the reported Tibetan variants in the first exon of *EGLN1*, c.12C > G (Asp4Glu) is absent and 380G > C (Cys127Ser) is found at low frequency in Andeans from Cerro de Pasco, Peru (Heinrich et al., 2019), further supporting the occurrence of different adaptive mechanisms despite an overlap in the adaptive genetic signal. Preliminary epigenetic investigation of specific sites in the *EGLN1* region in Andeans suggests distinct levels of methylation between Andeans with and without excessive erythrocytosis (Julian, 2017; Julian and Moore, 2019), and recent studies show variants at this locus are associated with exercise capacity in Andeans (Brutsaert et al., 2019). More than 30 other genes were reported by Bigham et al. (2010), including *EDNRA* and *PRKAA1*, which were both associated with birthweight and the latter with metabolic homeostasis in subsequent genotype-phenotype analysis (Bigham et al., 2014). Several additional genes reported in these studies have since been reported as adaptive in other Andean populations or identified in other highland populations (e.g., the beta hemoglobin (*HBB*) cluster region and *EDNRA*) (Simonson, 2015; Supplementary Table S1).

Additional studies of Andean adaptation suggest genes related to cardiac function, rather than hypoxia responses, are essential for survival in this population. Cardiac-related genes *VEGFB* and *ELTD1* were identified within the strongest regions of selection in the Colla population from the Argentinian Andes (Eichstaedt et al., 2014). Analysis of low-coverage whole-genome sequence highlighted a distinct set of genes related to cardiovascular function (*BRINP3*, *NOS2*, and *TBX5*) and suggested these genes, rather than those in hypoxia-response pathways, are the strongest targets of selection in Andeans (Crawford et al., 2017).

Another whole-genome analysis based on analyses of Andeans with and without CMS reported *SENP1* and *ANP32D* as top targets for adaptation (Zhou et al., 2013), and gene expression in fibroblasts is lower in cells derived from individuals without compared to those with CMS (Zhou et al., 2013). Additional studies based on single nucleotide polymorphism (SNP) microarray analysis indicate *FAM213A* and *SFTPD* (Valverde et al., 2015), are associated with oxidative stress and respiration and innate defenses, respectively, in Quechua and Aymara populations.

Using a new statistical approach to detect very recent positive selection, Eichstaedt et al. (2017) compared whole-genome sequences from high-altitude Argentinians and lowland Native Americans and identified a missense variant in the *EPAS1* gene, one of the major adaptive genes reported in Tibetans, and another in *GPR126*, which was associated with lung function (Eichstaedt et al., 2017). This is the first study to report *EPAS1* as adaptive in Andean populations and, like *EGLN1*, suggests different variants play pivotal roles in each population’s adaptive genetic profile.

### Human Genetic Adaptation to High Altitude: Ethiopian Highlanders

Highland Ethiopians also exhibit distinct physiological and genetic adaptations to high altitude, although they have been less studied than their Tibetan and Andean counterparts. The population history of Ethiopian highlanders is complex, due to repeated migrations into and out of the Ethiopian highlands spanning the past 70, 000 years (Hassen, 1990). Several genomic studies published thus far provide important insights into convergence of the HIF as well as distinct adaptive pathways (Scheinfeldt et al., 2012; Bigham and Lee, 2014; Petousi and Robbins, 2014; Simonson, 2015; Moore, 2017).

Putatively adaptive copies of the *THRB* gene region as well as *PPARA* and *EPAS1* identified in Tibetans (Simonson et al., 2010) show relationships with hemoglobin concentration in Amhara Ethiopians (Scheinfeldt et al., 2012). *EDNRB* (endothelial receptor B), previously reported as a top selection candidate in Andeans (Bigham et al., 2010), is also reported in Amhara Ethiopians, and knockdown of this gene increases hypoxia tolerance in mice (Udpa et al., 2014). The gene family member *EDNRA* is also a top candidate gene in Tibetans (Simonson et al., 2010). *BHLHE41*, although not associated with hemoglobin, is a key HIF pathway gene and top selection candidate in Amhara, Oromo, and Tigray Ethiopians (Huerta-Sánchez et al., 2013). In addition to these hypoxia-associated genes, three others (*VAV3*, which encodes vav guanine nucleotide exchange factor 3, and *RORA* that encodes the RAR-related orphan receptor A), are reported as top candidates in Amhara Ethiopians (Scheinfeldt and Tishkoff, 2013). Whole-genome sequence analyses indicate three genes contained within the same region of chromosome 19 identified as adaptive targets in Oromo and Simen Ethiopians, *CIC*, *LIPE*, and *PAFAH1B3* (that encode capicua transcriptional repressor, lipase E hormone-sensitive type, and platelet-activating factor acetylhydrolase 1b catalytic subunit 2, respectively) have orthologs in *Drosophila* that afford tolerance to hypoxia (Udpa et al., 2014). Comparison of genome-wide epigenetic profiles from saliva samples collected in high and low altitude Oromo Ethiopians indicated differences at several genes (Alkorta-Aranburu et al., 2012).

While various candidate genes are highlighted due to replication and/or association with phenotype, hundreds of distinct putatively adaptive gene regions have been identified and may or may not be reported in individual studies to date. Therefore, inconsistencies among studies may reflect differences in analytical approaches and reporting, variation within a continental region, and/or the stage and degree of adaptation. Additional efforts to standardize analysis within and across continental populations are needed to fully understand the extent of overlap among humans.

Many genes originally highlighted as adaptive targets in human high-altitude studies have also emerged as top candidates for genetic adaptation in other species under various conditions of environmental hypoxia. For example, as in high-altitude human populations, adaptations involving hematological values are found in most hypoxia-adapted high-altitude animal populations. Specifically, high-O_2_ affinity hemoglobin is found in many highland animals, including alpacas (Piccinini et al., 1990), Andean and bar-headed geese (Jessen et al., 1991; Zhang et al., 1996; Liang et al., 2001), deer mice (Storz et al., 2007, 2009), and yaks (Weber et al., 1988) (among others), and also in many lowland animals that are adapted to hypoxia, such as naked mole-rats (Johansen et al., 1976). Despite major overlap in genetic pathways (i.e., the hemoglobin gene cluster and associations with blood-O2 affinity and the hypoxia HIF pathway genes, e.g., *EPAS1* and *EGLN1*), different variants with putatively distinct functions are reported as targets of selection among highland human and other animal populations.

More recent analyses of whole genome sequences (e.g., in Andeans, Zhou et al., 2013; in Tibetans, Hu et al., 2017) indicate that non-protein coding variants, including those in heterochromatic or DNA methylated portions of the genome, are crucial for adaptation. In such cases, the effects of increased or decreased gene expression could vary across tissues and/or stages of development (in contrast to protein-coding variants that result in uniform alterations across all cells). An understanding of these fine-tuned, context-specific changes could provide much needed insight into molecular mechanisms of adaptation that influence genetic pathways in ways that are similar or not to other hypoxia-adapted species.

## Animals

### Domesticated High-Altitude Mammal Populations

As humans spread across the globe and to altitude, they brought a variety of domesticated mammals for companionship, labor, defense, and food. As a result, these animals have undergone strong selection for the tasks they perform for their human masters (e.g., for size, etc.), and so analysis of genetic adaptations to altitude in these populations must take this into account. Nonetheless, the study of domesticated high-altitude mammal populations provides an opportunity to contrast adaptive strategies to hypoxia between relatively short- and long-term (on a generational scale) exposures to low oxygen environments (Witt and Huerta-Sanchez, 2019).

One good example of such anthropocentric-driven short-term adaptation to chronic hypoxia in domesticated mammals can be found in feral horses of the Andean Paramo. This region of the Andes is a challenging environment with large temperature, humidity, and rainfall variance, high radiation, and minimal food availability. As a result, ungulates that had naturally evolved to live in this niche have largely shifted their range to more temperate regions. Conversely, populations of feral horses, which were originally introduced by Spanish conquistadores in the 1500’s, have thrived in this niche and the success of these introduced horses over the past ∼200 generations provides an important opportunity to evaluate genetic changes driven by this relatively short-term population-history exposure to hypoxia at altitude. Importantly, genes in Andean feral horse populations can be easily compared to those of their lowland Iberian ancestors (Hendrickson, 2013). A recent genomic analysis between these two populations revealed a mutation in *EPAS1* in the high-altitude population. Specifically, BIEC2– 310909 (rs69041973) is highly different between the lowland and highland species, although this SNP is intronic and has no known function. Unfortunately, little is known regarding physiological adaptations to hypoxia in this species and so it is difficult to draw direct comparisons between human studies in this case; however, the commonality of the occurrence of mutations in HIF-related genes in high-altitude populations is important to note and will be a recurring theme in this section.

Another useful study model are dogs, whose range has expanded in step with their human companions since the paleolithic era (Germonpre et al., 2009). On the Tibetan plateau, Tibetan mastiffs, which are derived from the native lowland Chinese Native dog, have undergone human-driven selection for life at high altitude (Li and Zhang, 2012). As a result, these mastiffs have lower hemoglobin levels than do Chinese native dogs (Wen and Yuan, 1998), and genome analysis revealed a mutation in *EPAS1* and also in *SIRT7*, which negatively regulates HIF1 and HIF2 (Li et al., 2014). The frequency of specific variants within these genes scale with altitude, as demonstrated by a second study that compared the genomes of five dog species whose range spans continuous altitudes along the Tea Horse Road in the Tibetan Plateau and a European dog species as an out-group (Gou et al., 2014). In this analysis, four novel non-synonymous mutations in *EPAS1* were found between the high-altitude dog population and both the mid- and low-altitude populations studied. Furthermore, all remaining genes that varied in this comparison are regulated by the HIF pathways, including the *HBB* cluster region, similar to Tibetan and Andean human populations. These researchers also examined hematological properties in these species and found that the high-altitude populations had a lower peripheral vascular resistance, which would enhance the flow of blood otherwise made more viscous by higher hemoglobin concentrations at altitude.

Intriguingly, and as we see in high-altitude human populations, this adaptation in high-altitude adapted dogs may be due to accelerated adaptations through admixture: i.e., the spread of beneficial alleles between closely related species. In this case, there is evidence that mutations to *EPAS1* in high-altitude dogs is due to admixture from interbreeding with Tibetan wolves (VonHoldf et al., 2017). The gray wolf is the most widely distributed terrestrial mammal with as many as 32 sub-species. One of these is the Tibetan gray wolf (*Canis lupis chanco*), which is endemic to the Tibetan plateau (Aggarwal et al., 2003). Genetic comparison between the Tibetan gray wolf and low-altitude wolf populations in China revealed positive selection of hypoxia-related genes in HIF-signaling pathways, including three SNPs unique to the highland populations in *EPAS1*, two in *RYR2*, and one in *ANGPT1* (Zhang W. P. et al., 2014). Along with *EPAS1*, *ANGPT1* functions in the HIF pathway and can increase vascularization and thereby oxygen delivery (Prabhakar and Semenza, 2012). *ANGPTL4* functions in this same gene pathway is reported as an adaptive gene candidate in Tibetans (Simonson et al., 2010). A recent study examined the degree of admixture between Tibetan mastiffs and wolves at the *EPAS1* gene and reported an excess of highland gray wolf ancestry at the *EPAS1* locus in the highland domestic dogs (VonHoldf et al., 2017). This finding suggests that an adaptive variant of *EPAS1* was transferred from wolves to dogs through interbreeding, thereby accelerating the adaptation to high altitude in the later species through adaptive introgression.

A similar adaptive history is apparent in analysis of Tibetan sheep (*Ovis aries*), which were also brought to altitude as a domesticated animal by humans [∼ 3, 100 years ago (Hu et al., 2019)]. Contrary to in dogs, higher hemoglobin concentration is observed in high altitude-resident populations of Tibetan sheep relative to lowland populations, along with higher hematocrit (Wei et al., 2016) (although peripheral vascular resistance has not been examined in this species and adaptations here may compensate for increased blood viscosity). These adaptations are associated with shorter-term life histories at altitude. However, positive selection has occurred in the form of 12 mutations to the *EPAS1* gene in Tibetan sheep (Wei et al., 2016). Importantly, this study also examined the effect of several splice variants of *EPAS1* on hematological parameters and determined that at least one mutation was associated with a gain of hemoglobin concentration. This divergent physiological phenotype, despite evolutionary pressure on the same gene, highlights the complexity of relating physiological and genetic studies of adaptation to high altitude. Interestingly, as in dogs, the changes in the sheep genome may be due to adaptive introgression from local species, in this case from argali (*Ovis ammon*), which may have accelerated the adoption of mutations to commonly targeted genes (Hu et al., 2019), similar to reports of high-altitude dog populations.

Another interesting model, and one of the largest resident mammals on the Tibetan Plateau, are yaks (*Bos grunniens*). Yaks currently exist in both domesticated and wild populations on the plateau, but which were initially brought to altitude ∼ 4, 500 years ago by humans (Meyer et al., 2009; Qi et al., 2013; Qiu et al., 2015). Relative to other high-altitude mammals, the physiology of yaks has been reasonably well-characterized, and adaptations to hypoxia in this species include an increased pulmonary surface area, reduced gas diffusion barriers, lower hemoglobin, and larger lungs, than are found in lowland cattle (Qi et al., 2019). Yaks at sea level and at altitudes of 2260−4500 m exhibit lower pulmonary artery pressures (Ishizaki et al., 2005). Genomic analysis of yaks indicates several HIF-specific mutations have occurred in high *vs*. low altitude populations, including to two important regulators of HIF (*ADAM17* and *ARG2*) (Qiu et al., 2012, 2015; Guang-Xin et al., 2019). Furthermore, transcriptomic analysis of yaks endemic to an altitudinal gradient demonstrated that *EPAS1* expression increases with altitude (Qi et al., 2019). Intriguingly, genomic analysis of Tibetan cattle also suggests introgression of adaptive mutations driven by life in hypoxia due to hybridization with yaks (Wu et al., 2018). Clearly, this is a common route of accelerating adaptive mutations in domesticated species brought to high-altitude by humans.

### Wild High-Altitude Animal Populations

At the other end of this spectrum are species that have lived at high altitude for hundreds of thousands of years in the wild (and/or that have been domesticated by high altitude human populations but not transported to high altitude). One example species are Tibetan wild boars, which live at ∼ 4300m. A recent genome comparison between this species and the European domestic Duroc pig revealed 13 positively selected genes in the “response to hypoxia” category (Li et al., 2013), of which several are downstream targets of HIF pathways, including *FIGF*, *PGF*, and *VEFPC*. A similar phenotype is found in Tibetan pigs, which are native to the Tibetan Plateau and inhabit a niche ranging in altitude from 2, 900 to 4, 300 m. Intriguingly, and similarly to adapted Tibetan human populations, Tibetan pigs have a blunted erythropoietic response to high-altitude hypoxia and have lower hemoglobin concentrations relative to lowland pigs at sea level or acclimated to high-altitude (Ai et al., 2014). At the genetic level, genome analysis revealed that Tibetan pig populations (after considering various sub populations and back flow of genes from low altitude populations) exhibit a loss of function mutation in *EPAS1* (Ai et al., 2013, Ai et al., 2014; Li et al., 2013). In addition, another study demonstrated that mutations to an intron 5′-CGTG-3′ sequence of *TMPRSS6* increase with increasing residence altitude in Tibetan pigs (Kong et al., 2019). *TMPRSS6* is a downstream gene of the HIF pathway that is associated with serum iron concentration and hemoglobin levels (Chambers et al., 2009; Tanaka et al., 2010). Hemoglobin also scaled with altitude in this study (Kong et al., 2019). The 5′-CGTG-3′ intron sequence is associated with binding to HREs (Camenisch et al., 2001), suggesting that HIF modulation of blood properties is under Darwinian selection with increasing altitude in this species. This mutation thus likely contributes to blunted erythropoietic response to hypoxia in these pigs.

An opposing hematological phenotype is found in Tibetan cashmere goats (*Capra hircus*), which are one of the more ancient domesticated species in Tibet and have the broadest altitudinal range of Chinese herbivores, spanning from sea level to the top of the Tibetan Plateau (∼ 0 – 5,000 m) (Wang et al., 2011). Unlike many large high-altitude mammals, some physiological data is available regarding their adaptations to altitude relative to lowland populations: highland populations have a higher hemoglobin concentration and a lower resting heart rate (Huang, 1980; Renzheng and Ciren, 1992). This is in striking contrast to previous studies in Tibetan human and pig populations, wherein hemoglobin concentrations are lower relative to non-adapted human populations but blood flow is higher. Here, genetic analysis revealed a missense mutation in *EPAS1* which results in an amino acid substitution in HIF-2α at a site adjacent to its dimerization interface with HIF-1β (Wang et al., 2011; Song et al., 2016). This is intriguing and suggests that mutations to *EPAS1* may result in either an up or down-regulation of HIF-related signaling pathways. This divergence in gene-driven physiological function, which is dependent upon the site of mutation, may be a key determinant of adaptation *vs*. maladaptation to high altitude in various animal and human populations.

Another ungulate, the Tibetan antelope (*Pantholops hodgsonii*), is a particularly athletic species found at elevations of ∼4, 000−5, 000 m. A recent genomic study examined conserved genetic changes between this species and two species of high-altitude pika: the American Pika (*Ochotona princeps*), and the Tibetan Pika (*O. curzoniae*) (Ge et al., 2013). This study identified seven genes (*ADORA2A*, *CCL2*, *ENG*, *PIK3C2A*, *PKLR*, *ATP12A*, and *NOS3*) as being under positive selection in all three high-altitude species. Of particular interest is *NOS3*, which influences nitric oxide production and thereby blood vessel diameter. In addition, *PKLR* is a top candidate of selection in Tibetans (Simonson et al., 2010, 2012; Yi X. et al., 2010).

One of the best studied high-altitude mammals is also one of the smallest. The plateau zokor (*Myospalax baileyi*) inhabits the Tibetan plateau and dwells in underground nests, in which the ambient oxygen level is likely even lower than that at the surface of the plateau (Zhou and Dou, 1990). Relative to lowland rodents, plateau zokors have high hemoglobin and hematocrit, and a low heart rate (Wei and Ma, 2001; Wei et al., 2006). A genetic comparison between this species and the Norwegian rat (*Rattus norvegicus*) revealed positively selected genes in the blood vessel development category, including *ANGPTL3*, *PRCP*, *SEC24B*, *EPHA1*, *MCAM*, *KAT6A*, *MYH9*, *CYSLTR2*, *MYLK*, *SPINT1*, *PPAP2B*, and *STRA6* (Shao et al., 2015). Most of these genes are under regulation by the HIF pathway. In addition, evaluation of parallel evolution between the plateau zokor and the naked mole-rat (see below) revealed several genes related to hypoxia that were similarly modified in both hypoxia-tolerant species, including *EPAS1*, *SLC29A1*, *MYO5B*, *AJUBA*, *TGFBR3*, *VASN*, *ACAA2*, *PINK1*, *SIRT1*, *SIRT2*, *SOD2*, and *NARFL*. Of particular interest is that *EPAS1* mutations in both naked mole-rats and plateau zokors are identical. *AJUBA* is also a key regulator of HIF function (Bridge and Sharp, 2012) and is similarly mutated in both species (Shao et al., 2015). Furthermore, transcriptome analysis between different populations of Zokors endemic to increasing elevations indicated that mutations to *EPAS1*, *COX1*, and *EGLN1* all scaled with increasing altitude (Cai et al., 2018).

### Birds

Many avian species live in high-altitude regions and/or transverse high-altitude regions in their annual migrations (Altshuler, 2006; Bishop et al., 2015). In addition to the hypoxic and hypothermic challenges faced by other species at altitude, birds face additional challenges during active flight related to low air density and cold air (Sun et al., 2016). Some birds that are resident to high-altitude environments compensate for these challenges with increases in wing size and stroke amplitude (Altshuler et al., 2004; Sun et al., 2016); however, this comes with the added cost of greater body mass and a higher metabolic rate, which in turn enhances energetic challenges in hypobaric hypoxia. Thus, the lifestyles of different bird species likely have a large effect on the evolution of adaptations to life at high-altitude.

Perhaps the best example of an avian species endemic to high altitude with high exercise requirements are apex avian predators, such as raptors. On the Tibetan Plateau, this is the saker falcon (*Falco cherrug*), which colonized this region as recently as 2000 years ago (Pan et al., 2017). Little is known regarding physiological adaptations to hypoxia in this species, but a recent study compared the genomes of falcon populations across Eurasia at three elevations (lowland, steppe, and Tibetan Plateau) and found a strong positive exonic selection signal on the *EPAS1* gene (Pan et al., 2017), despite this very short colonization time at altitude. Hemoglobin was also altered in this comparison, and transcriptomic analyses revealed that 50% of the transcripts (6/12) that were upregulated in the saker falcon were related to hypoxia response or hematopoiesis. Here, transcripts that are regulated by *EPAS1* transcription were also elevated in the saker falcon and transcripts whose expression levels correlated with that of *EPAS1* were almost uniformly elevated (21/24 transcripts).

Another avian population of interest are Andean ducks, of which there are many species that include both low and high-altitude populations, making these an excellent model for comparison. A recent study examined convergent evolution at the HIF pathway between high and low elevation populations of the yellow-billed pintail (*Anas georgica*), the cinnamon teal (*A. cyanoptera*), and the speckled teal (*A. flavirostris flavirostris/A. f. oxyptera*) (Graham and McCracken, 2019). This study revealed strong support for convergent evolution on the HIF pathway, with common mutations found in the high-altitude populations on the exonic regions of *EPAS1* and the intronic regions of *ELGN1*.

An interesting alternative model are flightless birds. For example, the Tibetan ground tit (*Parus humilis*), which is a largely ground-bound bird that is endemic to the Tibetan Plateau and is only found above the treeline (i.e., above 3, 300 m) (Qu et al., 2013). Genetic analysis of the Tibetan ground tit revealed that the majority of gene ontology (GO) categories modified in this species [relative to the closely related great tit (*P. major*) and yellow-cheeked tit (*P. spilonotus*)], were associated with increased energy metabolism (Qu et al., 2013), presumably to support heat production in this cold climate. Furthermore, and similar to Tibetan human populations, some genes related to hypoxia are also under positive selection in the ground tit, including the HIF-1 alpha subunit inhibitor (*HIF1AN*), *ANGP4 (ANGPTL4)*, which is involved in angiogenesis, and *ADAM9 (ADAMTS9)*, which is an important regulator of HIF1 (Qiu et al., 2012; Qu et al., 2013). Each of these is reported within the top 2% of selection candidate genes in Tibetans (Simonson et al., 2010). Interestingly, *EPAS1* was not under selection in this avian species, at least in this comparison.

Similarly, the Tibetan chicken (*Gallus gallus*), which is an aboriginal breed found on the Tibetan plateau, is flightless and adapted to life at high altitude. Compared to low altitude chickens, the Tibetan chicken is smaller, and has higher hematocrit and hemoglobin concentration and also a higher hemoglobin-oxygen affinity (Zhang et al., 2007). A recent study sequenced and compared SNPs of Tibetan and lowland chickens to test for potential variations in *EPAS1* in these species (Li et al., 2017), and this analysis revealed two SNPs in *G. gallus EPAS1* that correlate with high-altitude adaptation.

### Ectotherms

A key consideration in evaluating genetic changes in animals and human populations that are resident to high altitude is that such environments also incorporate numerous powerful evolutionary drivers in addition to hypoxia. For example, reduced atmospheric protection at altitude results in increased UV exposure and radiation (Blumthaler et al., 1997); annual and daily temperature and humidity swings are also more pronounced at high altitude than at lower altitudes. As a result, it can be difficult to filter out genetic changes that underlie adaptations to life in hypoxia from genetic changes that are due to other selective pressures. This limitation can be partially overcome by studying ectothermic species that live at high altitude, in which endogenous thermoregulatory complications endemic to life at elevation may be removed from consideration. Toward this aim, the genomes of three Tibetan hot-spring snakes (*Thermophis*) were recently sequenced and compared to those of lowland ectotherms (Li J. T. et al., 2018). Not surprisingly, this study identified three shared amino acid replacement mutations in the *EPAS1* gene of hot-spring snakes that are consistent with a downregulation in function relative to that of the lowland species. One of these mutations was in the DNA-binding domain of *EPAS1*, and this mutation limited erythropoietin expression in human 293T cells when transfected using a plasmid approach. This study indicates that common mutations in the HIF-signaling architecture have occurred in response to the adaptive pressure of high-altitude hypoxia in both resident endothermic and ectothermic species and that, similar to the other studies discussed above, point mutations in *EPAS1* may have up or down-regulating effects on physiological adaptations.

Another ectothermic species of interest is the toad-headed sand lizard (*Phrynocephalus erythrurus*), which typically live at altitudes of >4, 500 m and are generally considered to be the most high-altitude-adapted lizard known (Yang Y. Z. et al., 2015). A comparative analysis between the genomes of this species and that of a lower-altitude adapted congener (*P. putjatia*) revealed that *UBE2D1* was positively selected for in the high-altitude population (Yang Y. Z. et al., 2015). This gene enables the ubiquitination of HIF1 alpha. Not surprisingly, the hemoglobin concentration and capillary density of *P. erythrurus* is greater than in the lowland cousin (He et al., 2013). Another study comparing lowland and highland populations of toad-headed sand lizards (*P. przewalskii* and *P. vlangalii*, respectively) found similar results (Yang et al., 2014). Specifically, *ADAM17* was positively selected in the high-altitude population, and so was *MB*, which encodes for myoglobin. Once again, in this comparison the high-altitude population expresses higher hemoglobin concentration and hematocrit than the lowland population (He et al., 2013). Interestingly, a similar analysis of a pair of ranid frog species, including the high-altitude dwelling *Rana kukunoris* and the low-altitude dwelling *R. chensinensis* similarly revealed positive selection on *UBE2D1*, suggesting convergent evolution at this site in ectotherms adapted to hypobaric hypoxia (Yang Y. Z. et al., 2015).

### Fish

As is clear from the studies reviewed above, the study of genomic adaptations to life at high altitude has largely focused on endothermic species and a small number of ectothermic vertebrates. However, there are also numerous aquatic species that are native to highland bodies of water and fish in these niches are beginning to draw attention as model systems in which to study adaptations to life at altitude. To date, no study has examined genomic adaptation in a highland fish but the genomes of two fish species that are endemic to the Tibetan plateau – *Glyptosternon maculatum* and *Trilophysa tibetana* – have been recently sequenced with an eye toward future use of these genomes to evaluate mutations associated with life at high altitude (Liu et al., 2018; Yang et al., 2019).

### Summary of Common Gene Targets in High-Altitude Animal Populations

It is clearly apparent that life in hypobaric hypoxia at altitude has driven genetic and physiological adaptations related to hematological parameters in almost all species studied to date. Similarly, this evolutionary pressure appears to have modified angiogenesis, which is largely initiated by the HIF pathway (Rey and Semenza, 2010), suggesting that vascular changes are a crucial response to life in chronic hypoxia. Indeed, candidate genes that contribute to angiogenesis and/or vasculogenesis have been detected in numerous species living at high altitudes, although there is little overlap in specific genes among species. For example, positive selection of *SRF*, *TXNRD2*, and *WNT7B* were detected in the ground tit (Qu et al., 2013), *NOS3* was detected in the Tibetan antelope (Ge et al., 2013), and *ADAM17* was detected in both the pig (Ai et al., 2013), toad-headed lizard (Yang et al., 2014), yak (Qiu et al., 2012), and humans (Simonson et al., 2010), and all modify vascularity. These findings suggest the potential occurrence of convergent evolution along multiple genetic pathways toward a common physiological phenotype in high-altitude adapted populations, although additional studies are required to link genotypes to phenotypes. As we will see in the next section, hypoxia in lowland niches has driven similar adaptations. An extensive table of genes associated with high-altitude adaptation shared among humans and other hypoxia-adapted species, along with references, can be found in Supplementary Table S1.

### Hypoxia-Tolerant Species Endemic to Low Altitudes

Beyond studies in high-altitude resident ectotherms, studies in species that are native to low altitude hypoxic environments are perhaps more useful in teasing out genetic adaptations that commonly arise as a result of hypoxia specifically, from those related to damage repair (e.g., following radiation) or thermoregulation. Indeed, hypoxia and hypothermia often have opposing effects on development, with the former favoring small body size and low metabolic rates and the later favoring the opposite. Therefore, physiological and genetic studies of high-altitude populations may be confounded by these opposing evolutionary drivers, clouding the impact of hypoxia-alone on genetic mutations and physiological adaptations. Fortunately, hypoxic environments are commonly found beyond high altitude niches. For example, underground burrows and frozen lakes and ponds and are lowland niches in which many hypoxia-tolerant species are found, across the animal kingdom. A key caveat in this comparison, however, is that lowland hypoxic environments are likely not consistently or uniformly hypoxia; denizens of these niches likely experience gradients of hypoxia and even periods of normoxia in their day-to-day activities. This periodic intermittent hypoxic exposure results in divergent stresses to which these animals must adapt (e.g., reoxygenation injury, hypercapnia) and therefore likely exerts a different selection pressure than life in hypobaric hypoxia; the effects of normobaric and hypobaric hypoxia further remain unclear and require additional standardized analyses (Coppel et al., 2015). However, genomic analysis where available has revealed genetic mutations that in many cases are common in their target to those found in high-altitude adapted animal populations.

Of particular interest are naked mole-rats (*Hetercephalus glaber*), which are among the most hypoxia-tolerant mammal presently identified and tolerate minutes of complete anoxia, hours at 3% O_2_, and days to weeks at 8% O_2_ (Pamenter et al., 2015; Chung et al., 2016; Park et al., 2017). Naked mole-rats are endemic to north eastern Africa and are not found at high-altitudes. However, they live in large colony groups and putatively experience severe hypoxia in their crowded nest chambers due to the combination of a large number of animals breathing in small space and poor gas diffusion through the surrounding soil. A recent genome study of this species revealed mutations in the VHL binding domain (Kim et al., 2011), which results in a high endogenous expression of HIF protein in this species. Perhaps not surprisingly, naked mole-rats exhibit a hemoglobin-oxygen affinity that is similar to that of neonatal rodents (Johansen et al., 1976). These observations highlight that chronic hypoxia may not be required to drive the physiological and genomic mutations that are commonly found in high-altitude vertebrate populations.

As mentioned above, genes in the HIF pathway have been targets of selection across multiple species. How these variants relate to phenotypes within and across species will provide import insight into the primary and secondary effects of adaptations and maladaptation to hypoxia (Storz and Scott, 2019) and require detailed functional assessments (Hall et al., 2020).

## Future Directions

From studies of high-altitude adapted human and animal populations it has become abundantly clear that life in hypobaric hypoxia has driven the convergent evolution of specific genetic pathways across very disparate animals. In species that have lived at altitude for shorter periods of time, admixture from other species that have lived at altitude for longer has played a key role in accelerating these adjustments. Intriguingly, the occurrence of hematological adaptations that enhance blood flow seems to be a common theme at both the physiological and genetic levels, which are easily studied across species. In recent years, genetic analyses have in some ways outpaced physiological studies as high-throughput population-wide approaches become more widely utilized. However, the physiological impact of many of the mutations identified in high-altitude populations and species are unknown, and it is highly likely that adaptations beyond hematological function will prove to be important in tolerating hypoxia at altitude. Indeed, it is very likely that adaptations at the metabolic and cellular levels are critical to tolerating life in hypoxia. Conversely, numerous studies have examined physiological adaptations to hypoxia in a systemic context in a wide variety of comparative animal species; however, genetic information is lacking for many of these species. As such, the next major step in this field will be to marry genetic, omic, and physiological knowledge to fully elucidate beneficial physiological adaptations to hypoxia and their evolutionary origins.

## Author Contributions

MP and TS designed the report. All authors produced figures and drafted and contributed comments to the final manuscript.

## Conflict of Interest

The authors declare that the research was conducted in the absence of any commercial or financial relationships that could be construed as a potential conflict of interest.
