# Supplementary material for: Cross-Species Insights Into Genomic Adaptations to Hypoxia
Source: Front Genet. 2020 Jul 22;11:743. doi: 10.3389/fgene.2020.00743 (PMC7387696; doi:10.3389/fgene.2020.00743)
Supplement: Supplementary file 1 [file Table_1.DOCX]

**Table S1. Genes associated with hypoxic adaption among humans and other species.**

| Candidate gene region | Tibetan/Sherpa, Andean, Ethiopian | Other intermediate/highland populations | Phenotype association in human highland population | Hypoxia-adaptive significance in non-human species |
| --- | --- | --- | --- | --- |
| *EPAS1* | Tibetan^1-9^, Sherpa^10^, Andean^11^ | Deedu Mongolian^12^ | Hemoglobin concentration in (Tibetan^2,5^, Amhara Ethiopian^13^); lactate, free fatty acids (Tibetan^14^) | Tibetan Dog^15-19^ ,Tibetan Grey Wolf^15,16,19^, Tibetan horse^20^ , Andean Horse^16,20^, Tibetan sheep^21^, Yak^22^, Tibetan pig^23-26^, Tibetan wild boar^24^, Tibetan goats^27,28^, Plateau Zokors^29,30^,  Naked Mole Rat^29^, Tibetan Saker Falcon^31^, Tibetan Chicken^32^, Tibetan Hot-spring Snake^33^, Yellow-billed Pintail, Cinnamon Teal, and Speckled Teal^34^ |
| *EGLN1* | Tibetan^1,4-9,35,36^, Andean^3,37^, Sherpa^10^ | Daghestani^38^ | Hemoglobin concentration (Tibetan^1^); Gain^39^ and loss^40,41^ of function in exon 1 Asp4Glu/Cys127Ser | Plateau Zokors^30^, Yaks^22^, Tibetan wild boar^24^, Yellow-billed Pintail, Cinnamon Teal, and Speckled Teal^34^ |
| *PPARA* | Tibetan^1^, Amhara and Omotic Ethiopian^13^ |  | Hemoglobin concentration (Tibetan^1^, Amhara and Omotic Ethiopian^13^); lactate, free fatty acids (Tibetan^14^) |  |
| *HMOX2/NMRAL1* | Tibetan^1,4,8^ |  |  |  |
| Hemoglobin Beta gene region | Tibetan^1,5^, Andean^3^ |  |  | Deer Mice^42,43^,  Hummingbird^44^,  Dog^15,18^ |
| *PKLR* | Tibetan^1,5^ | Deedu Mongolian^12^ |  | Tibetan Pika^45^ |
| *CYP17A1* | Tibetan^1,8^ |  |  |  |
| *HFE* | Tibetan^1,5,8^ |  |  |  |
| *EDNRA* | Tibetan^1^  Andean^3^ |  | Birth weight (Andeans^46^) |  |
| *CYP2E1* | Tibetan^1^ | Deedu Mongolian^12^ |  |  |
| *PPARG* | Tibetan^1^ | Deedu Mongolian^12^ |  |  |
| *HYOU1/HMBS* | Sherpa^10^ |  | Hemoglobin concentration (Sherpa^10^) |  |
| *SENP1/ANP32D* | Andean^47,48^ |  |  | *Drosophila^47^* |
| *ADAM17* | Tibetan^1^ |  |  | Yak^22,49^, Toad-headed Sand Lizards^50^, Tibetan Pig^23^ |
| *ARNT2, CBARA1, THRB, VAV3* | Amhara Ethiopian^13^ |  | *THRB* and hemoglobin concentration (Amhara Ethiopian^51^) |  |
| SNP rs10803083 (chromosome 1) | Amhara Ethiopian^51^ |  | Hemoglobin concentration in Amhara Ethiopian^51^ |  |
| *BHLHE41* | Amhara, Oromo, and Tigray Ethiopian^52^ |  |  |  |
| *PRKAA1* | Andean^37^ |  | Birth weight (Andeans^37,46^); Maternal genotypes associated with the uterine artery diameter and metabolic homeostasis (Andeans^46^) |  |
| *EDNRB* | Andean^3^, Amhara Ethiopian^14^ |  |  |  |
| *CIC, LIPE, PAFAH1B3* | Amhara/Oromos Ethiopian^14^ |  |  | Involved in hypoxia tolerance in *Drosophila*^14^ |
| *NOS1* | Sherpa^53^ |  |  | Plateau Zokors^30^, Tibetan ducks^34^, Yaks^22^, Tibetan pigs^26^ |
| *ARG2* |  |  | Skeletal muscle development^54^ | Tibetan pigs^54^,  Tibetan goat^28^, Tibetan wild boar^24^, Yaks^22,49^ |
| *RYR2* |  |  | Upregulated in hypoxic injury^55^ | Tibetan Grey Wolf^15^, Tibetan pigs^26^, Tibetan wild boar^24^, Tibetan chicken^56^ |
| *HIF1A* | Tibetan^6^, Andean^57^ |  | Skeletal muscle development^54^; Master regulator of hypoxic signaling^6^ | Plateau Zokors^30^, Yaks^22^, Tibetan wild boar^24^ |
| *SOD2* |  |  | Positive feedback between HIF1a and SOD2^58^ | Andean horse^20^, Plateau Zokors^30^, Yaks^22^ |
| *ABAT** |  |  |  | Andean horse^20^, Yaks^22^, Tibetan wild boar^24^, Tibetan frogs^59^, Adzuki bean weevil^60^ |
| *MTOR* |  |  |  | Plateau Zokors^30^, Tibetan ground tit^61^, Tibetan ducks^34^ |
| *ACACB* |  |  |  | Tibetan pigs^26^, Yaks^22^, Tibetan wild boar^22,24^ |
| *PAPSS2* |  |  |  | Tibetan pigs^26^, Yaks^22^, Tibetan Goat^28^ |
| *LEP* |  |  |  | Plateau Zokors^30^, Tibetan wild boar^24^, High altitude dogs^15^ |
| *CCL2* |  |  |  | Plateau Zokors^30^, Yaks^22^, Tibetan antelope^45^ |
| *MMP2* |  |  |  | Plateau Zokors^30^, Tibetan wild boar^24^, Yaks^22^ |
| *TGFB1* |  |  |  | Plateau Zokors^30^, Tibetan wild boar^24^, Yaks^22^ |

**REFERENCES**

1 Simonson, T. S. *et al.* Genetic evidence for high-altitude adaptation in Tibet. *Science* **329**, 72-75, doi:10.1126/science.1189406 (2010).

2 Beall, C. M. *et al.* Natural selection on EPAS1 (HIF2alpha) associated with low hemoglobin concentration in Tibetan highlanders. *Proc. Natl. Acad. Sci. U. S. A.* **107**, 11459-11464, doi:10.1073/pnas.1002443107 (2010).

3 Bigham, A. *et al.* Identifying signatures of natural selection in Tibetan and Andean populations using dense genome scan data. *PLoS Genet.* **6**, e1001116, doi:10.1371/journal.pgen.1001116 (2010).

4 Peng, Y. *et al.* Genetic variations in Tibetan populations and high-altitude adaptation at the Himalayas. *Mol. Biol. Evol.* **28**, 1075-1081, doi:10.1093/molbev/msq290 (2011).

5 Yi, X. *et al.* Sequencing of 50 human exomes reveals adaptation to high altitude. *Science* **329**, 75-78, doi:10.1126/science.1190371 (2010).

6 Xu, S. *et al.* A genome-wide search for signals of high-altitude adaptation in Tibetans. *Mol. Biol. Evol.* **28**, 1003-1011, doi:10.1093/molbev/msq277 (2011).

7 Wang, B. *et al.* On the origin of Tibetans and their genetic basis in adapting high-altitude environments. *PloS one* **6**, e17002, doi:10.1371/journal.pone.0017002 (2011).

8 Wuren, T. *et al.* Shared and unique signals of high-altitude adaptation in geographically distinct Tibetan populations. *PloS one* **9**, e88252, doi:10.1371/journal.pone.0088252 (2014).

9 Buroker, N. E. *et al.* EPAS1 and EGLN1 associations with high altitude sickness in Han and Tibetan Chinese at the Qinghai-Tibetan Plateau. *Blood Cells Mol. Dis.* **49**, 67-73, doi:10.1016/j.bcmd.2012.04.004 (2012).

10 Jeong, C. *et al.* Admixture facilitates genetic adaptations to high altitude in Tibet. *Nat. Commun.* **5**, 3281, doi:10.1038/ncomms4281 (2014).

11 Eichstaedt, C. A. *et al.* Evidence of Early-Stage Selection on EPAS1 and GPR126 Genes in Andean High Altitude Populations. *Sci. Rep.* **7**, 13042, doi:10.1038/s41598-017-13382-4 (2017).

12 Xing, J. *et al.* Genomic analysis of natural selection and phenotypic variation in high-altitude mongolians. *PLoS Genet.* **9**, e1003634, doi:10.1371/journal.pgen.1003634 (2013).

13 Scheinfeldt, L. B. *et al.* Genetic adaptation to high altitude in the Ethiopian highlands. *Genome Biol.* **13**, R1, doi:10.1186/gb-2012-13-1-r1 (2012).

14 Udpa, N. *et al.* Whole genome sequencing of Ethiopian highlanders reveals conserved hypoxia tolerance genes. *Genome Biol.* **15**, R36, doi:10.1186/gb-2014-15-2-r36 (2014).

15 Gou, X. *et al.* Whole-genome sequencing of six dog breeds from continuous altitudes reveals adaptation to high-altitude hypoxia. *Genome Res.* **24**, 1308-1315, doi:10.1101/gr.171876.113 (2014).

16 vonHoldt, B., Fan, Z., Vecchyo, D. O.-D. & Wayne, R. K. EPAS1 variants in high altitude Tibetan wolves were selectively introgressed into highland dogs. *PeerJ* **5**, e3522, doi:10.7717/peerj.3522 (2017).

17 Wang, G.-D. *et al.* Genetic Convergence in the Adaptation of Dogs and Humans to the High-Altitude Environment of the Tibetan Plateau. *Genome Biology and Evolution* **6**, 2122-2128, doi:10.1093/gbe/evu162 (2014).

18 Li, Y. *et al.* Population variation revealed high-altitude adaptation of Tibetan mastiffs. *Mol. Biol. Evol.* **31**, 1200-1205, doi:10.1093/molbev/msu070 (2014).

19 Miao, B., Wang, Z. & Li, Y. Genomic Analysis Reveals Hypoxia Adaptation in the Tibetan Mastiff by Introgression of the Grey Wolf from the Tibetan Plateau. *Molecular Biology and Evolution*, msw274, doi:10.1093/molbev/msw274 (2016).

20 Hendrickson, S. L. A genome wide study of genetic adaptation to high altitude in feral Andean Horses of the páramo. *BMC Evol. Biol.* **13**, 273, doi:10.1186/1471-2148-13-273 (2013).

21 Wei, C. *et al.* Genome-wide analysis reveals adaptation to high altitudes in Tibetan sheep. *Sci. Rep.* **6**, 26770, doi:10.1038/srep26770 (2016).

22 Qi, X. *et al.* The Transcriptomic Landscape of Yaks Reveals Molecular Pathways for High Altitude Adaptation. *Genome Biol. Evol.* **11**, 72-85, doi:10.1093/gbe/evy264 (2019).

23 Ai, H., Huang, L. & Ren, J. Genetic diversity, linkage disequilibrium and selection signatures in chinese and Western pigs revealed by genome-wide SNP markers. *PloS one* **8**, e56001, doi:10.1371/journal.pone.0056001 (2013).

24 Li, M. *et al.* Genomic analyses identify distinct patterns of selection in domesticated pigs and Tibetan wild boars. *Nat. Genet.* **45**, 1431-1438, doi:10.1038/ng.2811 (2013).

25 Li, M. *et al.* Reply to 'On genetic differentiation between domestic pigs and Tibetan wild boars'. *Nature Genetics* **47**, 192-192, doi:10.1038/ng.3193 (2015).

26 Ai, H. *et al.* Population history and genomic signatures for high-altitude adaptation in Tibetan pigs. *BMC Genomics* **15**, 834, doi:10.1186/1471-2164-15-834 (2014).

27 Wang, Y. *et al.* Genetic diversity of Tibetan goats of Plateau type using microsatellite markers. *Archives Animal Breeding* **54**, 188-197, doi:10.5194/aab-54-188-2011 (2011).

28 Song, S. *et al.* Exome sequencing reveals genetic differentiation due to high-altitude adaptation in the Tibetan cashmere goat (Capra hircus). *BMC Genomics* **17**, 122, doi:10.1186/s12864-016-2449-0 (2016).

29 Shao, Y. *et al.* Genetic adaptations of the plateau zokor in high-elevation burrows. *Scientific Reports* **5**, doi:10.1038/srep17262 (2015).

30 Cai, Z. *et al.* Adaptive Transcriptome Profiling of Subterranean Zokor, Myospalax baileyi, to High- Altitude Stresses in Tibet. *Sci. Rep.* **8**, 4671, doi:10.1038/s41598-018-22483-7 (2018).

31 Pan, S. *et al.* Population transcriptomes reveal synergistic responses of DNA polymorphism and RNA expression to extreme environments on the Qinghai-Tibetan Plateau in a predatory bird. *Mol. Ecol.* **26**, 2993-3010, doi:10.1111/mec.14090 (2017).

32 Li, S. *et al.* A non-synonymous SNP with the allele frequency correlated with the altitude may contribute to the hypoxia adaptation of Tibetan chicken. *PloS one* **12**, e0172211, doi:10.1371/journal.pone.0172211 (2017).

33 Li, J.-T. *et al.* Comparative genomic investigation of high-elevation adaptation in ectothermic snakes. *Proc. Natl. Acad. Sci. U. S. A.* **115**, 8406-8411, doi:10.1073/pnas.1805348115 (2018).

34 Graham, A. M. & McCracken, K. G. Convergent evolution on the hypoxia-inducible factor (HIF) pathway genes EGLN1 and EPAS1 in high-altitude ducks. *Heredity* **122**, 819-832, doi:10.1038/s41437-018-0173-z (2019).

35 Ge, R.-L. *et al.* Metabolic insight into mechanisms of high-altitude adaptation in Tibetans. *Mol. Genet. Metab.* **106**, 244-247, doi:10.1016/j.ymgme.2012.03.003 (2012).

36 Xiang, K. *et al.* Identification of a Tibetan-specific mutation in the hypoxic gene EGLN1 and its contribution to high-altitude adaptation. *Mol. Biol. Evol.* **30**, 1889-1898, doi:10.1093/molbev/mst090 (2013).

37 Bigham, A. W. *et al.* Identifying positive selection candidate loci for high-altitude adaptation in Andean populations. *Hum. Genomics* **4**, 79-90, doi:10.1186/1479-7364-4-2-79 (2009).

38 Pagani, L. *et al.* High altitude adaptation in Daghestani populations from the Caucasus. *Hum. Genet.* **131**, 423-433, doi:10.1007/s00439-011-1084-8 (2012).

39 Lorenzo, F. R. *et al.* A genetic mechanism for Tibetan high-altitude adaptation. *Nat. Genet.* **46**, 951-956, doi:10.1038/ng.3067 (2014).

40 Song, D. *et al.* Defective Tibetan PHD2 binding to p23 links high altitude adaption to altered oxygen sensing. *J. Biol. Chem.* **289**, 14656-14665, doi:10.1074/jbc.M113.541227 (2014).

41 Sinnema, M. *et al.* Loss-of-function zinc finger mutation in the EGLN1 gene associated with erythrocytosis. *Blood* **132**, 1455-1458, doi:10.1182/blood-2018-06-854711 (2018).

42 Storz, J. F. *et al.* Evolutionary and functional insights into the mechanism underlying high-altitude adaptation of deer mouse hemoglobin. *Proc. Natl. Acad. Sci. U. S. A.* **106**, 14450-14455, doi:10.1073/pnas.0905224106 (2009).

43 Natarajan, C. *et al.* Epistasis among adaptive mutations in deer mouse hemoglobin. *Science* **340**, 1324-1327, doi:10.1126/science.1236862 (2013).

44 Projecto-Garcia, J. *et al.* Repeated elevational transitions in hemoglobin function during the evolution of Andean hummingbirds. *Proc. Natl. Acad. Sci. U. S. A.* **110**, 20669-20674, doi:10.1073/pnas.1315456110 (2013).

45 Ge, R.-L. *et al.* Draft genome sequence of the Tibetan antelope. *Nat. Commun.* **4**, 1858, doi:10.1038/ncomms2860 (2013).

46 Bigham, A. W. *et al.* Maternal PRKAA1 and EDNRA genotypes are associated with birth weight, and PRKAA1 with uterine artery diameter and metabolic homeostasis at high altitude. *Physiol. Genomics* **46**, 687-697, doi:10.1152/physiolgenomics.00063.2014 (2014).

47 Zhou, D. *et al.* Whole-genome sequencing uncovers the genetic basis of chronic mountain sickness in Andean highlanders. *Am. J. Hum. Genet.* **93**, 452-462, doi:10.1016/j.ajhg.2013.07.011 (2013).

48 Cole, A. M., Petousi, N., Cavalleri, G. L. & Robbins, P. A. Genetic variation in SENP1 and ANP32D as predictors of chronic mountain sickness. *High Alt. Med. Biol.* **15**, 497-499, doi:10.1089/ham.2014.1036 (2014).

49 Qiu, Q. *et al.* The yak genome and adaptation to life at high altitude. *Nat. Genet.* **44**, 946-949, doi:10.1038/ng.2343 (2012).

50 Yang, W., Qi, Y. & Fu, J. Exploring the genetic basis of adaptation to high elevations in reptiles: a comparative transcriptome analysis of two toad-headed agamas (genus Phrynocephalus). *PloS one* **9**, e112218, doi:10.1371/journal.pone.0112218 (2014).

51 Alkorta-Aranburu, G. *et al.* The genetic architecture of adaptations to high altitude in Ethiopia. *PLoS Genet.* **8**, e1003110, doi:10.1371/journal.pgen.1003110 (2012).

52 Huerta-Sánchez, E. *et al.* Genetic Signatures Reveal High-Altitude Adaptation in a Set of Ethiopian Populations. *Molecular Biology and Evolution* **30**, 1877-1888, doi:10.1093/molbev/mst089 (2013).

53 Zhang, C. *et al.* Differentiated demographic histories and local adaptations between Sherpas and Tibetans. *Genome Biology* **18**, 115, doi:10.1186/s13059-017-1242-y (2017).

54 Zhang, J., Chen, L., Long, K. R. & Mu, Z. P. Hypoxia-related gene expression in porcine skeletal muscle tissues at different altitude. *Genet. Mol. Res.* **14**, 11587-11593, doi:10.4238/2015.September.28.10 (2015).

55 Kesherwani, V. & Agrawal, S. K. Upregulation of RyR2 in Hypoxic/Reperfusion Injury. *Journal of Neurotrauma* **29**, 1255-1265, doi:10.1089/neu.2011.1780 (2012).

56 Wang, M.-S. *et al.* Genomic Analyses Reveal Potential Independent Adaptation to High Altitude in Tibetan Chickens. *Mol. Biol. Evol.* **32**, 1880-1889, doi:10.1093/molbev/msv071 (2015).

57 Rupert, J. L. *et al.* Genetic polymorphisms in the Renin-Angiotensin system in high-altitude and low-altitude Native American populations. *Ann. Hum. Genet.* **67**, 17-25, doi:10.1046/j.1469-1809.2003.00004.x (2003).

58 Gao, Y.-H. *et al.* Hypoxia-inducible factor 1α mediates the down-regulation of superoxide dismutase 2 in von Hippel–Lindau deficient renal clear cell carcinoma. *Biochemical and Biophysical Research Communications* **435**, 46-51, doi:10.1016/j.bbrc.2013.04.034 (2013).

59 Yang, W., Qi, Y., Bi, K. & Fu, J. Toward understanding the genetic basis of adaptation to high-elevation life in poikilothermic species: a comparative transcriptomic analysis of two ranid frogs, Rana chensinensis and R. kukunoris. *BMC Genomics* **13**, 588, doi:10.1186/1471-2164-13-588 (2012).

60 Cui, S. F. *et al.* Comparative transcriptome analyses of adzuki bean weevil (Callosobruchus chinensis) response to hypoxia and hypoxia/hypercapnia. *Bull. Entomol. Res.* **109**, 266-277, doi:10.1017/S0007485318000512 (2019).

61 Qu, Y. *et al.* Ground tit genome reveals avian adaptation to living at high altitudes in the Tibetan plateau. *Nat. Commun.* **4**, 2071, doi:10.1038/ncomms3071 (2013).
